# Biphasic Computational Fluid Dynamics Modelling of the Mixture in an Agricultural Sprayer Tank

**DOI:** 10.3390/molecules25081870

**Published:** 2020-04-18

**Authors:** Jorge Badules, Mariano Vidal, Antonio Boné, Emilio Gil, F. Javier García-Ramos

**Affiliations:** 1 Escuela Politécnica Superior, University of Zaragoza, 22071 Huesca, Spain; jbadules@hotmail.com (J.B.); vidalcor@unizar.es (M.V.); anbone@unizar.es (A.B.); 2 Department of Agri-Food Engineering and Biotechnology, Universitat Politécnica de Catalunya, Castelldefels, 08860 Barcelona, Spain; emilio.gil@upc.edu

**Keywords:** spray tank, agitation system, product concentration, Euler-Euler models, plant protection products

## Abstract

Agitation inside agricultural sprayer tanks can be studied while using an international standard procedure, based on obtaining internal samples of liquid. However, in practice, this test is not easy to perform. Herein, we propose the explicit study of the mixing procedure with biphasic computer simulations using Computational Fluid Dynamics (CFD). An experimental test was performed on a 3000 L tank of a commercial air-assisted sprayer, with two different agitation system configurations, in order to compare the results of several theoretical physical models of biphasic flows for CFD, both Eulerian and Lagrangian. From the analysis of these theoretical models, we conclude that the Volume of Fluid model is not viable and the Discrete Phase Model produces erroneous results, while the Eulerian and Mixture models can both be useful. However, the results obtained suggest that complex streams generated by real-world agitation systems produce more errors in calculations. Both models can be conducted in the design phase, prior to the implementation of the machine. In addition, the computer simulations allow for researchers to analyse the mixing process in detail, making it possible to evaluate the efficiency of an agitation system according to the time that is required to reach mixture homogeneity.

## 1. Introduction

Agricultural pesticides are widely used to fight against fungi, insects, and weeds. These products are applied with different types of sprayers, but their use generally requires dissolution in a water tank at the manufacturer recommended concentration.

These tanks have agitation systems that move the liquid to achieve homogeneity and prevent sedimentation. Standard requirements [1] for sprayer design (including Venturi agitators, if necessary) guarantee this homogeneity with a maximum deviation of 15%. There is also a standard procedure for studying agitation, the ISO 5682-2 standard [2]. After introducing a tracer to the solution, this procedure requires taking samples at three different levels after 10 min of agitation, and then repeating the process 16 h later. In practice, this procedure is time-consuming and labor intensive. As an alternative, several methods have been proposed to more efficiently study agitation in sprayer tanks, including the use of turbidity meters [3,4], photography of transparent tanks [5], glass microsphere sedimentation studies [6], and correlating the homogeneity of the solution with measurements of velocities in the liquid of the tank [7].

Ensuring that the agitation system adequately fulfils its function is very important for the machine manufacturer; however, although some of the mentioned methodologies are simpler than the ISO standard [2], they all still require costly experimental measures to be added to previously manufactured equipment [8]. For this reason, numerical simulation prior to equipment development would allow for the efficiency of the agitation system to be evaluated in the design phase prior to manufacturing. To achieve this, Computational Fluid Dynamics (CFD) is a tool with great potential. This is a computer numerical calculation technique that solves problems involving fluids flows. Its most common application in the study of agricultural sprayer agitation systems has been the estimation of liquid velocities and the subsequent comparison with real values [9,10,11], the influence of different nozzle positions, orientations, and operating pressures [12], and the empirical study of concentration in the solution by correlating it with the velocities of a CFD model of a sprayer tank [13].

However, there are numerous studies that are based on CFD in the chemical industry that explicitly address the problem of mixture agitation in tanks by means of biphasic flow modelling. The most common case is the simulation of cylindrical tanks with solid-liquid mixtures, where agitation is performed with rotating impeller blades [14,15,16]. A study comparing the density of a mixture of different oils in a storage tank with corresponding CFD simulation highlights the usefulness of this approach for liquid-liquid mixing problems [17]. The simulation of liquid-gas mixtures is also possible [18]. However, studies of tank simulations in which the agitation system consists of nozzles [19], similar to the most common agitation system in agricultural sprayers, are much less common.

Therefore, this work aims to evaluate the potential of biphasic CFD modelling of a mixture inside a spray tank of a commercial orchard sprayer. The purpose is to obtain explicit information regarding the concentration of the dissolved product at any point of the tank. For this purpose, different CFD models have been analyzed, and empirical data validated the estimated product concentration results. The main objective of this research is to provide sprayer manufacturers with a useful and ready-to-use procedure to improve agitation system designs.

## 2. Results and Discussion

### 2.1. Experimental Results of Agitation

The experimental results of the tests (Table 1) showed that high homogeneity is achieved in the tank mixture, both with two and four agitators working, as the calculated averages deviated very little from the theoretical 4 g/L of dispersed phase concentration that was introduced into the tank.

### 2.2. Results of Agitation with CFD Models

After presenting the experimental results, the results that were obtained from the CFD simulations with the different multiphase models are presented and discussed.

#### 2.2.1. DPM

**Figure 1:**
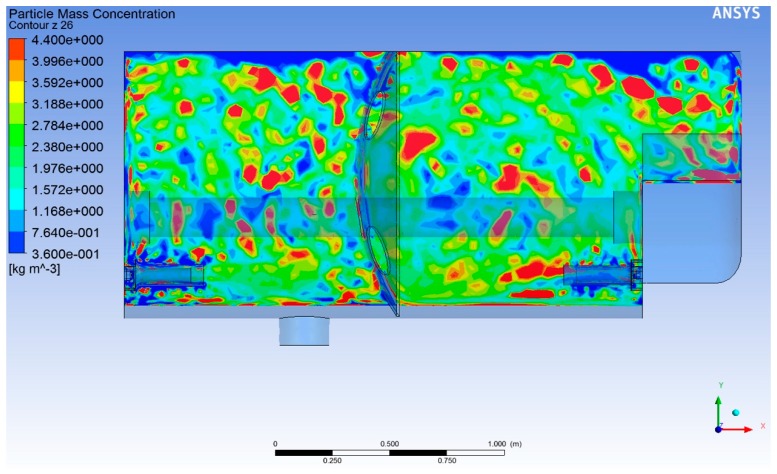
Concentration of the dispersed phase under the DPM model, with two working agitators, after 10 min of agitation simulation. The concentration results were not uniform. Lateral view of the tank. Concentration at a plane containing a pair of agitators.

Figure 1 shows the results of the tank agitation simulation using the DPM model with 3000 L, running two agitators, after 10 min of simulated time. Remarkably, the distribution of the secondary phase is not uniform at all, as would be expected from the experimental agitation results. The simulation shows that there were zones of the tank where the concentration was very low or non-existent, while in others the concentration was high, exceeding the range of the displayed scale. The calculation simulating four working nozzles provided similar results.

**Figure 2:**
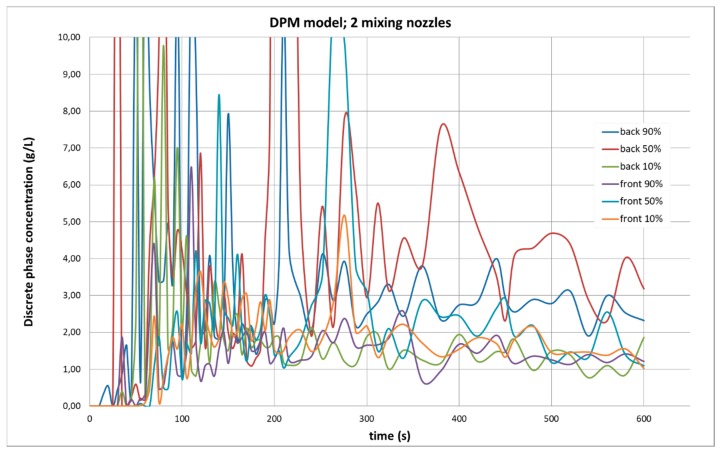
Evolution of the dispersed phase concentration during the simulation of agitation with the multiphase Discrete Phase Model (DPM) model, with two working nozzles. If the calculation were valid, an almost homogeneous concentration of 4 g/L should be obtained, which is the average amount of dispersed phase introduced in the calculation at the beginning.

The CFD simulations allow us to know the state of agitation in the computer model at any time. Figure 2 shows the evolution in the CFD simulation of the concentration at sampling points selected for the experimental measurements. When the simulation begins, the average concentration at the control points was zero, which is logical because the dispersed phase was introduced to the model in a confined zone of the tank far from the control points. Subsequently, the concentration presented volatile values in the first minutes of simulation, which are also explained by the movement of the dispersed phase through the tank when it was not yet mixed uniformly, so that, if a “cloud” of dispersed phase passed through a control point, the concentration increased violently, only to fall suddenly afterwards. However, although this behavior became smoother after the fifth minute of simulation (300 s), it never disappeared, even when the particles were already mixed throughout the model. The calculation at the end of the simulation was far from the expected one.

#### 2.2.2. Mixture Model

**Figure 3:**
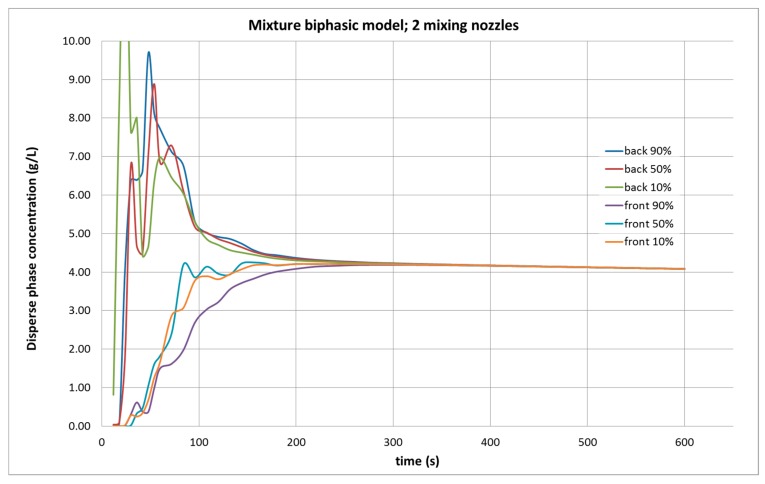
Evolution of the dispersed phase concentration in the Computational Fluid Dynamics (CFD) simulation of the tank under study, in a biphasic mixture model, with two agitators operating, from six control points in the front and the rear part of the tank, at 90, 50, and 10% of the height of water in the tank.

The results that were obtained with the Mixture model were utterly opposite. Although in the first minutes of the agitation simulation, the calculation indicated that the concentration of the product was very heterogeneous in the different control points; as time passed, the concentration converged to a single value. In the case of operating with two agitators, the concentration results are shown in the six control points (Figure 3) located in three different levels, in which the CFD programme predicts a very homogeneous mixture throughout the tank after about 300 s of simulation. In general, the simulation seems to be consistent with the experimental data, as the result of the simulation yields an average concentration of 4.08 g/L. That supposes a relative error of 2.5% with respect to the measured mean value of 3.98 g/L (Table 1).

**Figure 4:**
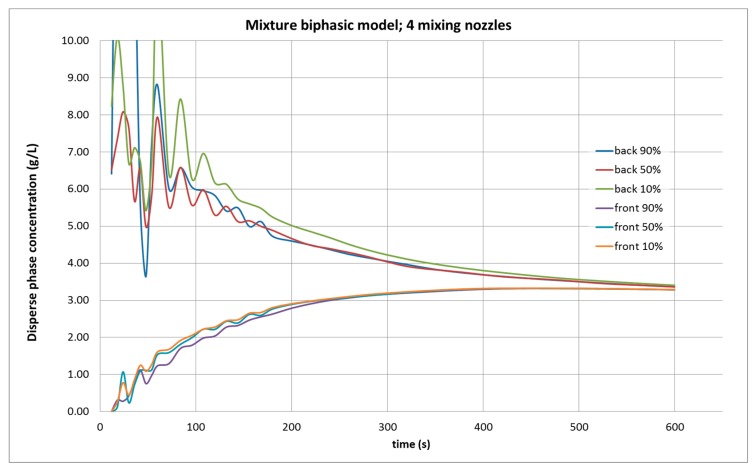
Evolution of the dispersed phase concentration in CFD simulation in the tank under study, with a biphasic mixture model, with four agitators operating, from six control points, in the front and the rear part of the tank, at 90, 50, and 10% of the height of water in the tank.

The results of the simulation with four agitators, as shown in Figure 4, predicted that 600 s were required to achieve homogeneity in the control points, which is much higher than the necessary time with only two nozzles running. In addition, the calculated average concentration is 3.3 g/L, well below the 3.97 g/L experimentally measured. A portion of the dispersed phase has been “lost” during this calculation.

#### 2.2.3. Eulerian Model

**Figure 5:**
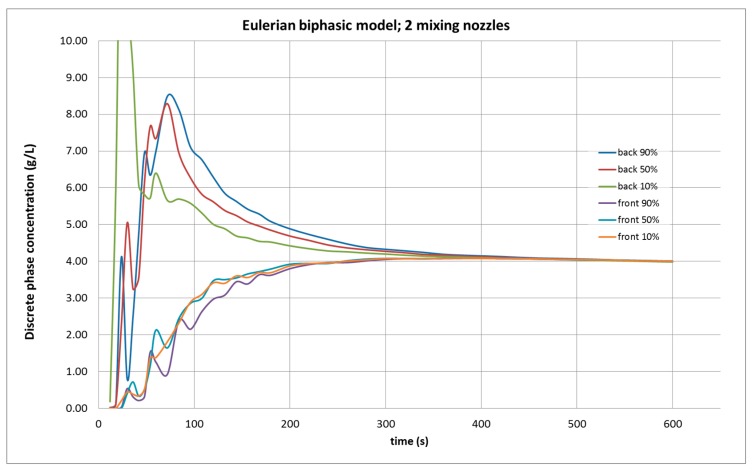
Evolution of the dispersed phase concentration in CFD simulation in the studied tank, under the biphasic Eulerian model, with two agitators working, from six control points.

Figure 5 shows the results of the agitation simulation with two agitators studied using the Eulerian biphasic model. The results were similar to the Mixture model, but the homogenization of the content in the tank required a longer time (about 100 s more). Experimentally, it is not possible to know which model reflects reality more accuracy, because continuous and real-time control of several points inside the tank cannot be realized with the methodology that was proposed by ISO [2].

Once homogeneity is reached in the tank during the last seconds of the simulation, the same trend was observed as in the previous Mixture model; the estimated CFD concentration decreases as the calculation progresses.

**Figure 6:**
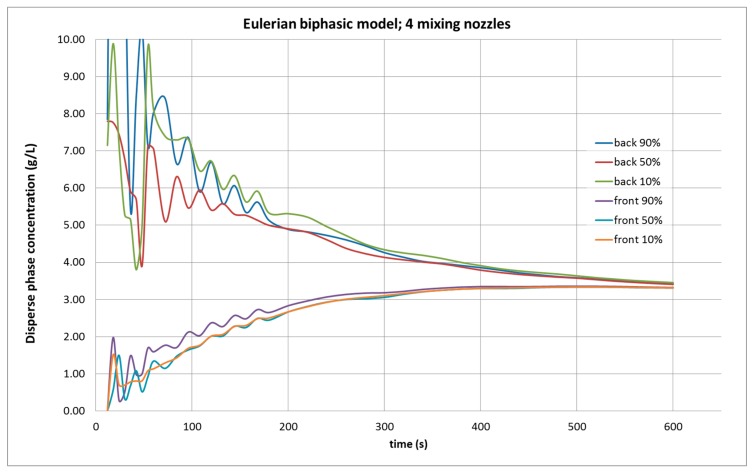
Evolution of the dispersed phase concentration in CFD simulation in the studied tank, under the biphasic Eulerian model, with four agitators working, from six control points.

The evolution of the concentration at control points for the Eulerian simulations was like those of the biphasic Mixture model, as shown in Figure 6. Numerically quantifying the results obtained in the simulations under the two-phase Mixture and Eulerian models, the data shown in Table 2 are obtained.

## 3. Discussion

The average concentration calculated for the six control points using the DPM model, as shown in Figure 2, was less than half the expected value at the end of the simulation. The calculation software itself offered part of the explanation, as, after 10 min of agitation simulation, the software indicated that it could not calculate the position of 2548 of the 60,000 sample particles studied. This is equivalent to 4.2% of the dispersed phase having been lost during the calculation, but it does not fully explain the losses, which were more than 50% in the control points. However, according to the CFD software, the average concentration calculated in the whole tank was 3.83 g/L. Therefore, the local concentration in certain areas of the model (Figure 1) had to be very high, which counteracted the low concentration regions in the average value calculation. The dispersed phase was not dissipated with this model, but it was not well distributed. We conclude from these results that multiphase agitation simulation using the DPM model leads to erroneous results.

Biphasic CFD simulation using the Eulerian model with two agitators is considered to be as successful, while the same average concentration calculation using the biphasic Mixture model was slightly above what was experimentally measured. Table 2 shows the predicted concentration values by the CFD simulation values and errors as compared with the experimental ones. When the four-agitator operation was simulated, both biphasic models followed the same pattern, but the Mixture model provided slightly lower values than the Eulerian one; however, neither model adequately matched the experimental results. Although both of the simulations did reflect the homogeneity of the mixture, the calculated average concentration value was lower than the experimental value. While a higher number of agitators in operation implies lower average fluid velocities within the tank, causing reduced agitation and lower concentrations of dispersed phase [7], the difference with the simulation is too significant to be explained by this phenomenon.

**Figure 7:**
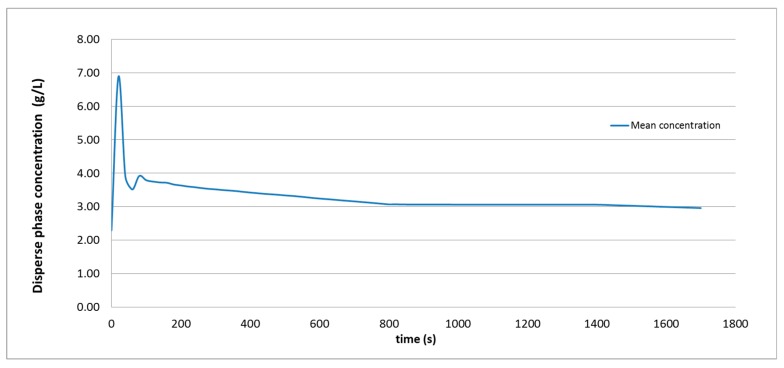
Evolution of the mean concentration during a simulation in which the calculation parameters are changed. Four agitators from t = 0 to t = 800 s, no agitation from t = 800 to t = 1400 s, two agitators after t = 1400 s.

Another cause must explain the errors compared to the experimental ones in the four agitator simulations. A new simulation with time-varying conditions was performed to understand these calculation discrepancies, as shown in Figure 7. From t = 0 to t = 600 s, the calculation was performed while considering the operation of four nozzles, simultaneously. From t = 600 s to t = 800 s, the four agitators continued to operate in the same way, but the calculation adopted the Reynolds Stress Model to simulate the turbulence, instead of the standard k-ε approach. Between t = 800 s and t = 1400 s, the agitation system was halted in the CFD simulation and the initial turbulence model calculation resumed. Finally, after t = 1400 s, the agitation system was activated again, but only with two active nozzles.

After an initial unstable phase, the concentration decreased at a constant rate, with no noticeable change due to the adoption of another turbulence model. The change could only be seen now when the agitation system stopped; from that moment, the dispersed phase concentration seems to remain constant. However, when the agitation system was activated again, with two nozzles, the dispersed phase decreased again, but at a much lower rate than in the stage with four nozzles in operation.

Therefore, the source of error cannot be attributed to either the mesh or the chosen turbulence model, which has also previously been shown [11] to provide reasonable results. We can also exclude the case of an inadequate time step, because a test simulation using four working agitators with 0.5 s time steps, in which the calculation residues of the dispersed phase were less than 1 × 10^−5^; the obtained results were very similar to those that were obtained with time steps of 1 s.

**Figure 8:**
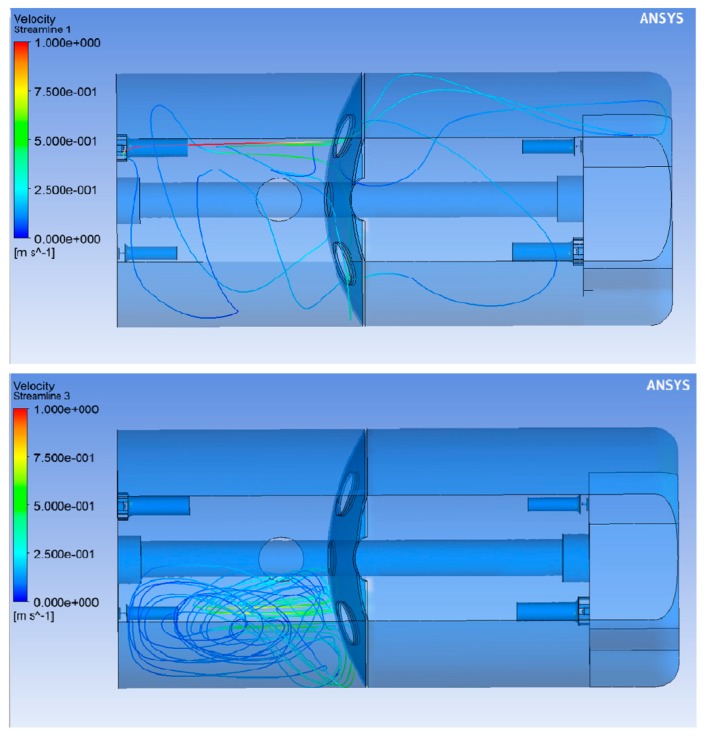
Top image of the tank with the main stream lines that pass through two points of the tank in front of two of the agitators. Top: only two agitators (those from opposite corners) are operating. Bottom: operation with all the agitators.

The significant error that accumulated in the calculation with four agitators might be because the streams generated in the tank (shown in Figure 8) are more complex (they involve more recirculation), so, under these conditions, the calculation errors in the dispersed phase were greater. The complex stream conditions have been studied previously [11], and they occur because the main jets collide in the breakwater holes when all of the agitators are activated, impeding an adequate exchange of fluid between the two sub-chambers of the tank. With two agitators operating, the flow runs freely through the whole tank, crossing the breakwater through its openings without any problem, as shown Figure 8; but with four agitators working simultaneously, the flows become independent in four internal recirculation cells.

## 4. Material and Methods

### 4.1. Apparatus and Experiment

**Figure 9:**
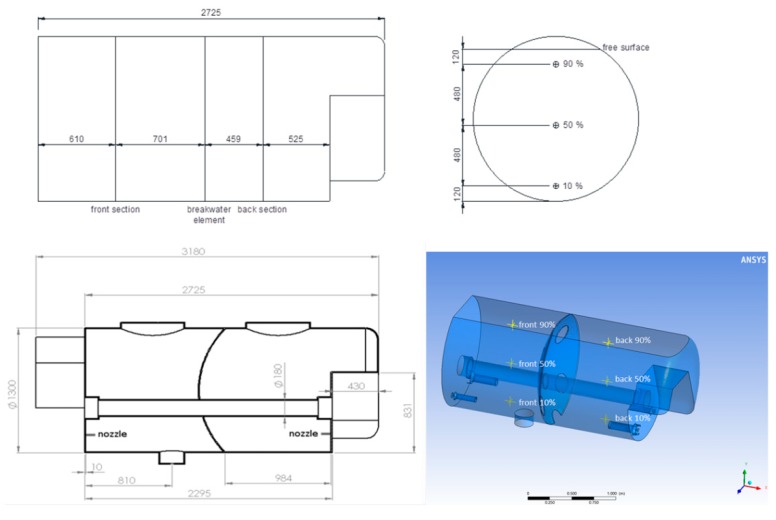
Location of sampling points (top); longitudinal cross-section of the tank (bottom left); three-dimensional (3D) scheme with sampling points (bottom right). Dimensions in mm.

An air-assisted sprayer with a 3000 L cylindrical geometry tank (GarMelet S.L., Zaidín, Huesca, Spain), with two interconnected cameras separated by a breakwater was used as a model sprayer. The tank has a cylindrical hole, through which a drive shaft passes across to activate the rear fan, according to Figure 9. There are four Venturi effect agitators inside the tank, which consist of two different models with different flowrates. These agitate the liquid and can operate either simultaneously or only the two highest flow agitators, which are placed at opposite corners.

A series of experimental measurements [7] and CFD modelling [11] have already been performed with this tank in relation to liquid velocities with different machine configurations. Accordingly, this work therefore extends previous studies, as the velocities of the liquid inside this tank had been studied. The results of these previous studies have been directly used in this work, as, for example, methodology for the meshing and certain configurations of the calculation. Additionally, in this research, biphasic CFD modelling of the mixture is implemented in such a way that the developed methodology would be extensible to the study of any agitation system of agricultural sprayers.

For this experiment, two different tests have been performed with the tank filled with 3000 L of water and the hydraulic circuit of agitation operating at 10-bar pressure:

Simultaneous use of two high-flow agitators only.

Simultaneous use of all four agitators, including two high-flow and two low-flow.

Once the tank was filled with 3000 L of clean water, 12 kg of copper oxychloride was added to achieve a theoretical average concentration of 4 g/L. After 10 min of agitation, samples were taken at three different levels of the tank: 10%, 50%, and 90% of the liquid height, located in a vertical line under each upper opening of the tank (front and back). Subsequently, the samples were dried, and the solid residue was weighed. Five samples were collected per point, giving a total of 30 samples per test.

### 4.2. CFD Models

For the resolution of all models, the software ANSYS-Fluent-17.0 (ANSYS, Inc., Canonsburg, PA, USA) was used as the CFD code. The problem under investigation consists of the computer modelling of a multiphase fluid, where the primary phase is water. In the context of the ANSYS-Fluent software, such multiple problems can approach using the four following mathematical models: Volume of Fluid (VOF), Eulerian, Mixture, and Discrete Phase Model (DPM). In the following sections, the advantages and disadvantages of each of these models for the problem under study are briefly described.

#### 4.2.1. VOF Model

The VOF model is an Euler-Euler model; that is, both primary and secondary phases are studied by means of an Eulerian approach. In the VOF model [20], the phases are immiscible. In our test case, the requirement for the Courant number to be less than 1, even in the most unfavorable cells [20] of the calculation mesh, is a major limitation of using this model. The Courant number is defined, as:

(1) $$C=\frac{v\times \Delta t}{\Delta x}$$

where *v* is the local velocity of the fluid, Δ*t* is the time step, and Δ*x* is the length of the edge of the local finite volume mesh cell. For our study, compliance with this condition is not feasible because the speed of the fluid is higher than 30 m/s in the injection zone of the nozzles, and the cell length is much less than one millimeter. The time step in the transient calculation would have to be so small (on the order of millionths of a second) that a conventional computer would take several years to simulate 1 min of agitation.

The use of this model was ruled out for this reason. It might be a suitable model for the study of tank agitation with rotor blades or propeller systems, because the maximum speed would be lower, but there are not many references [21] regarding its use for this purpose.

#### 4.2.2. Eulerian Model

The Eulerian model is also an Euler-Euler model, but with an important difference: the phases can be interpenetrating, i.e., the whole volume of a cell can be occupied simultaneously by several phases, as long as the sum of the phase percentages is one hundred.

The model represents a complex mathematical apparatus that makes it very computationally expensive, despite being the most widely used method [20] for studying highly heterogeneous multiphase flows, such as sedimentations or fluidized beds. Explicit compliance with the Courant condition, even in the most unfavorable cells, is not required.

#### 4.2.3. Mixture Model

The Mixture model is mathematically simpler than the Eulerian approach [20]; it is more stable and can be computed faster. This approach is more suitable for the study of multiphase flows in which the phases are moderately homogeneously distributed throughout the fluid. The Mixture and Eulerian models are the two models most frequently used to study the agitation of multiphase mixtures in the chemical industry.

#### 4.2.4. DPM Model

The DPM model is entirely different from the other three, as it is an Euler-Lagrange model; that is, although the primary phase is studied with a Eulerian approach, the secondary phases are numerically simulated while using a Lagrangian approach. In other words, the secondary phase is modelled as a collection of particles whose movement is calculated throughout the fluid. Rigorously speaking, it could be said that this is not a multiphase model but a monophasic one, to which the movement of discrete particles is coupled. It is not a very common model in the study of mixtures [22,23] when compared to the Euler-Euler model of interpenetrating phases.

Table 3 summarizes the characteristic differences between the models and their implementation in the present work.

#### 4.2.5. Meshing

CAD geometry of the tank was developed, which allowed for the simulation of a solid inside the tank representing the nominal capacity of the tank (3000 L). This action was complemented with the removal of the internal volume of the tank not occupied by the liquid. This solid was imported into a meshing module (Meshing-ANSYS ICEM CFD), where some initial meshing parameters were specified:

Unstructured type mesh

Minimum cell size 4.75 × 10^−4^ m

Maximum cell size 0.035 m

Six layers of additional mesh on the walls

Additionally, the boundary conditions were also defined. The liquid outlet was defined at the bottom of the tank. The orifices of the mixing nozzles were defined as inlets, while the physical limits of the tank were defined as wall. In order to define the boundary condition of the water-free sheet, which is not a wall and has no friction, or any phenomenon of viscous dissipation, it was defined as a zero flow fluid entry at atmospheric pressure. This strategy was previously used [11] with good results in this type of calculations.

**Figure 10:**
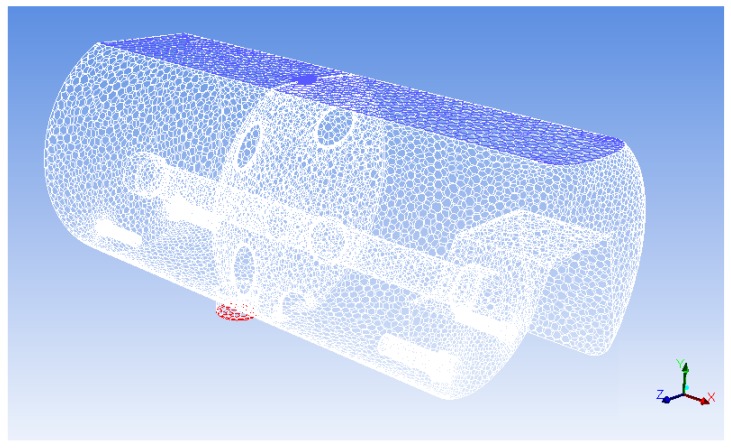
Mesh view in the ANSYS-Fluent software interface.

In this way, a mesh of more than one-million cells was obtained, which was imported into the CFD software, with which the final configuration was made. A simplified mesh of 122,609 cells (Figure 10) was obtained using the “polyhedral mesh” tool that incorporates the ANSYS-Fluent software, which was used to perform the calculation. This tool achieves by merging and changes in the geometry of the cells with the worst quality parameters, improving the quality of the mesh and reducing the number of cells. It was verified that this simplification offered similar results to the original mesh, thus saving calculation time.

As for the calculation, the Reynolds-Averaged Navier-Stokes (RANS) equations were solved with the standard k-ε turbulence model. While in CFD simulations of mechanically stirred tanks, this approach can present problems [16,21], for situations similar to the present work, it has been shown to be sufficiently reliable [15,19]. It is outside the scope of this paper to analyze turbulence with models other than RANS [24,25].

Scalable wall functions were implemented with the standard k-ε turbulence model, which have provided reasonable results in monophasic models [11] that were performed previously with the same tank. The spatial discretization methods used are the second-order upwind for momentum and turbulence model equations, QUICK for dispersed phase, and PRESTO for pressure.

### 4.3. Dispersed Phase in the CFD Model

The introduction of the dispersed phase is very different in the Euler-Euler models when compared to the Euler-Lagrange models. The DPM method implies the calculation of two independent, but coupled, phases, one dispersed and the other dispersant [20]. As a dispersed phase, inert particles are introduced. Any material can be modelled in the simulation as a dispersed phase, but the particle diameter must be congruent. It cannot cause rapid sedimentation of the particles, as that would be far from the conditions of the real experiment.

The introduction of 12 kg of secondary phase into the 3000 L model with particles of a few microns in diameter involves a huge number of particles (can be more than 10^10^). Although this would produce a very accurate model [26], it would be computationally intensive. The ANSYS-Fluent DPM method simplifies the calculation by limiting the number of particles and then extrapolating the behavior of the entire dispersed phase. Analogous to the study of meshing, it is convenient to perform a series of tests to determine what number of particles is reasonable to study [26]. This optimized number should be as large as possible without penalizing the calculation time and it should produce results that are similar to simulations with many more particles. In this way, it was determined that approximately 60,000 particles were sufficient. These particles were introduced at several points in a region of the mesh under the upper back cover and distributed over several seconds, so that large quantities do not accumulate in some cells. This prevents problems with the DPM method, which does not allow for a very high concentration of particles in any cell of the mesh. An appropriate boundary condition was imposed on the walls of the calculation domain, so that the particles could not escape the simulated tank.

The Euler-Euler models that were tested (both Eulerian and Mixture) handled the introduction of the dispersed phase differently. At the beginning of the experiment, a certain amount of secondary phase was set in a region of the mesh under the upper back cover (as in the previous case), where the products to be mixed in the tank are added.

All of the models required a fluid inlet (through the agitator nozzles) and an outlet (placed at the bottom outlet of the tank). As boundary conditions, the nozzles could not introduce a secondary phase from the outside and could not exit through the outlet (backflow = 0). The primary phase was the only product that entered and exited the finite volume model. This caused a certain distortion of the results in the proximity of the nozzles of the operating agitators and in the lower outlet of the tank, because the injected liquid comes from the recirculation of water from the tank and, therefore, does have dispersed phase. However, this strategy (not letting the secondary phase leave the tank) is justified because the dispersed phase concentration at the outlet in the CFD model is uncontrollable during the calculation.

### 4.4. Time Step and Convergence

The simulated models were intrinsically transient, so a time step between iterations had to be defined for the calculation, to understand the time evolution of the agitation. All of the models used accomplished with the Courant condition. The time step should ideally make the residuals of the calculation equations negligible (values less than 10^−4^). However, the calculated residuals reach values less than 10^−4^ reasonably quickly if a simple stationary monophasic calculation is performed, except for the continuity equation, which never reaches these values. It seems reasonable not to demand lower residuals in a transient problem than would be obtained in a stationary problem.

In this way, the criterion followed was first to solve the models in stationary mode, and then once the residuals of the continuity equation (the most unfavorable ones) had stabilized, a time step was chosen that provided residuals of the same order of magnitude in the transient calculations. In our case, a time step of 1 s was shown to be sufficient for the transient calculation.

## 5. Conclusions

The VOF model is not computationally viable with conventional computers, because meeting Courant’s condition requires the use of very small-time steps, so a simple simulation of several real minutes of agitation would take years of calculations. Furthermore, such a model is not suitable for this problem, because it is more appropriate for immiscible fluids.

The DPM model requires a limited number of particles to be introduced into the model and additional precautions to be taken in order to avoid divergence during calculation iterations. Even so, the obtained results were clearly wrong as compared to the ones measured experimentally, as the results did not conclude that the mixture was homogeneous.

The Eulerian and Mixture models provided satisfactory results. The results from both models were similar, so, in future simulations, we recommend using the Mixture model, because it requires less calculation time.

The study of biphasic flows in CFD seems to have one major limitation: the calculation fails when strong recirculating flows dominate. The error concerns not so much the general behaviour of the fluid, but mainly in the calculation of the average dispersed phase concentration, which deviates from the true value as the calculation advances. This deviation is apparently due to problems with rounding or interpolation of values within each iteration.

If a certain volume of clean water has been previously introduced into the tank, and the product to be applied to the crop is added, some time is required in order to achieve a homogeneous mixture. The CFD study of the agitation might indicate that one system or a configuration of agitators is better than another if less time is required to homogenize the mixture. Such conclusions cannot be obtained from the application of the ISO 5682-2 standard, giving the time-varying CFD models an advantage. In the tank that served as the basis for this study, it is preferable to use two nozzles instead of four, as the homogeneity of the mixture is clearly reached earlier with only two working nozzles.

Even when considering the encountered difficulties, the proposed method offers a new alternative for the established agitation test in actual standard. This proposal could serve sprayer manufacturers as an easy, cheap, and immediate useful results during the sprayer’s design.

## Figures and Tables

**Table 1:** Concentrations of the dispersed phase after 10 min of agitation, at 10%, 50%, and 90% of the liquid height in the tank. Mean and standard deviation of the samples taken at the back and front of the tank.

Configuration		Back		Front	
		Mean (g/L)	SD	Mean (g/L)	SD
Tow mixing nozzles	90%	3.975	0.011	4.001	0.024
	50%	3.991	0.015	3.955	0.051
	10%	3.993	0.043	3.96	0.041
Four mixing nozzles	90%	3.948	0.044	3.977	0.031
	50%	4.006	0.008	3.983	0.013
	10%	3.933	0.014	3.951	0.048

**Table 2:** Concentrations of the dispersed phase after 10 min of agitation, at 10%, 50%, and 90% of the liquid height in the tank. Mean and error in the control points in the CFD simulations with respect to the experimentally measured values. The results from biphasic Mixture and Eulerian models, at the back and front of the tank.

**Configuration**	**Mixture**				
		**Back**		**Front**	
		Mean (g/L)	Error (%)	Mean (g/L)	Error (%)
Two mixing nozzles	90%	4.086	−2.79%	4.081	−2.00%
	50%	4.083	−2.31%	4.08	−3.16%
	10%	4.08	−2.18%	4.087	−3.21%
Four mixing nozzles	90%	3.364	14.79%	3.284	17.43%
	50%	3.362	16.08%	3.284	17.55%
	10%	3.405	13.42%	3.289	16.76%
	**Eulerian**				
		**Back**		**Front**	
		Mean (g/L)	Error (%)	Mean (g/L)	Error (%)
Two mixing nozzles	90%	4.007	−0.81%	3.999	−0.55%
	50%	3.992	−0.03%	4.005	−0.55%
	10%	3.983	0.25%	4.002	−1.29%
Four mixing nozzles	90%	3.422	13.32%	3.322	16.47%
	50%	3.408	14.93%	3.311	16.87%
	10%	3.455	12.15%	3.312	16.17%

**Table 3:** Summary of the multiphase models, the secondary phase characteristics, and the model implemented in this research.

Model	Secondary Phase	Model Implemented
VOF	Immiscible	No. Calculation time unfeasible
Eulerian	Interpenetrating	Yes
Mixture	Interpenetrating (more mathematical simplicity than Eulerian)	Yes
DPM	Discrete particles	Yes
